# Editorial: Plant Silicon Interactions between Organisms and the Implications for Ecosystems

**DOI:** 10.3389/fpls.2016.01001

**Published:** 2016-07-15

**Authors:** Julia Cooke, Jane L. DeGabriel

**Affiliations:** ^1^Department of Environment, Earth and Ecosystems, The Open UniversityMilton Keynes, UK; ^2^Hawkesbury Institute for the Environment, Western Sydney UniversityPenrith, NSW, Australia

**Keywords:** plant silicon, biogeochemical cycles, herbivore defense, phytoliths, silica

Silicon (Si) is a beneficial, if not essential, plant nutrient (Epstein, 1994). As the second most abundant element in the Earth's crust it has a global cycling budget similar to that of carbon (Conley, 2002). Some ecological roles of Si are characterized (Cooke and Leishman, 2011), but recent technological advances mean unprecedented understanding of functions at multiple scales, and recognition of its importance to global biogeochemical cycling and food security. We present eight original research papers and an opinion article highlighting the novelty and diversity of recent research. New methods, fresh approaches in both applied and fundamental Si research, innovative herbivore defense experiments, ecosystem-scale field measurements, and Si changes under climate change are investigated. The diversity of topics reveals the complexity of plant responses in terms of Si accumulation, distribution, and function, which are contingent on genotype, biotic interactions, and environmental conditions.

High Si-accumulating plant species and families (especially Poaceae) have dominated Si research, including many articles in this research topic. Katz remind us that aside from Poaceae and economically important families such as Fabaceae and Cucurbitaceae, Si has functional roles in low Si-accumulating species. Katz argued that inter-familial variation among taxa is evidence of an ecologically important trait, and that research on low accumulators is likely to facilitate greater understanding of plant Si function.

Regardless of species, efficient methods for quantifying Si concentrations in plants are required. Smis et al. described a new approach to determine Si concentrations using near infrared reflectance spectroscopy (NIRS). They developed calibrations for predicting Si concentrations across diverse plant groups, with improved accuracy when models were restricted to a single species or family. The advantages of NIRS are that it is non-destructive, fast, and cheap, but it relies on robust calibrations from traditional laboratory analyses, such as X-Ray Fluorescence (Reidinger et al., 2012). Further work is required to standardize and improve techniques, but cost and time-effective procedures such as NIRS will surely facilitate ecological advances.

Silicon is added artificially in agriculture to reduce stress and improve production (Ma, 2004; Reynolds et al., 2009), which should be optimized and integrated into management. Keeping et al. examined impacts of Si, N, and water stress on two pests which reduce worldwide sugarcane production. Si addition significantly reduced borer damage, but did not impact the abundance of sucking thrips, perpetuating debate about how Si affects different herbivore types (Reynolds et al., 2009). Contrasting effects of Si on herbivores were found within a single plant, with higher Si resistance in stalks attacked by borers, but lower resistance in leaf spindles eaten by thrips.

Si accumulation can be induced by herbivory, but the trigger for and extent of the induced response is complex (McNaughton and Tarrants, 1983; Massey et al., 2007). Quigley and Anderson found Si induction was dependent on genotype and phylogeny, Si and water availability, grazing type and intensity, with water balance particularly important for some species. They showed consistently lower responses to artificial vs. real herbivory, contingent on damage frequency and intensity, consistent with other papers here (Huitu et al.; Hartley et al.).

Differences in induced Si accumulation and mechanisms of herbivore defense among and within plant species are poorly understood. Hartley et al. examined disparities in plants' ability to alter Si defenses in response to herbivory and Si supply. Species with similar total Si varied in leaf surface abrasiveness, because abrasive grasses had silicified spines, while others deposited silica in short cells. The quantity and distribution of Si was a complex interaction between genotype, Si availability, and herbivore damage. The authors argue that Si allocation to spines impacts palatability, while allocation to short cells may impact digestibility. Thus, there is no single anti-herbivore strategy of Si use by grasses.

Most studies consider Si defenses in isolation, but Huitu et al. examined interactions with another herbivore defense, endophytes (toxin-producing fungi). Tantalizingly, they demonstrated that Si concentrations were consistently ~16% higher in grasses with endophytes than those without, a similar magnitude to herbivore-induced Si uptake. This result was consistent across grazing levels with no interaction between grazing intensity, endophyte infection, and Si uptake detected. Endophytes could facilitate Si uptake and be beneficial against herbivory, although the higher Si uptake in endophyte-infected plants could also be a response to the intrusion of a foreign body.

Plant Si accumulation is complex, with aquaporins facilitating uptake in some species (Ma and Yamaji, 2015). Most studies have explored uptake using controlled experiments, but empirical data is needed to understand natural plasticity. At an ecosystem level, Carey and Fulweiler showed substantial natural variation in Si accumulation in *Spartina*, using new and published data. A conceptual model suggested that uptake is more plastic than previously recognized, driven by environmental factors and genetic origin. This challenges attributing a single Si accumulation value to a species and highlights that we lack a definitive method to characterize accumulation capacity.

Si weathers from primary silicates and cycles between plants and soils, eventually leaching into rivers and oceans. Cycling rate regulation is a hot topic and Li et al. investigated the dissolution potential of plant silica bodies (phytoliths) originating from different parts of rice plants. Phytoliths from leaves and sheaths were likely to contribute to faster recycling rates compared to grains and stems, linked to higher Si/Al and Si/Fe ratios. Genotype, morphology, and hydration rate also affected Si cycling. Consequently, variation in Si accumulation/deposition associated with herbivory, Si availability, and water balance described by others (Carey and Fulweiler; Quigley and Anderson; Hartley et al.) could have implications for Si cycling.

How plants Si accumulation and use might respond to climate change is unknown. Fulweiler et al. provide a first insight using data from a free-air CO_2_ enrichment experiment. They showed little change in foliar Si concentration under elevated atmospheric CO_2_. However, due to increased primary production, Carey and Fulweiler suggested that elevated atmospheric CO_2_ could significantly increase the Si pumping capacity of the vegetation in this system by up to 26%, with implications for C sequestration and downstream systems. Changes in transpiration rates under changing CO_2_ could also be a factor determining Si uptake in future climates.

Silicon concentration varies more than most elements among and within species (Epstein, 1999). This research topic presents novel advances in understanding the relationship between this variation and plant Si use. Factors determining accumulation are complex and often plastic, making further understanding an intriguing and promising challenge. An immediate challenge is understanding how global environmental change is likely to impact on Si in ecology and agriculture and the implications for ecosystem services and food security.

## Author contributions

JC and JLD are joint first authors of this paper, having equally contributed to the development of this research topic and its editorial.

### Conflict of interest statement

The authors declare that the research was conducted in the absence of any commercial or financial relationships that could be construed as a potential conflict of interest.
